# Whole-genome mutational burden analysis of three pluripotency induction methods

**DOI:** 10.1038/ncomms10536

**Published:** 2016-02-19

**Authors:** Kunal Bhutani, Kristopher L. Nazor, Roy Williams, Ha Tran, Heng Dai, Željko Džakula, Edward H. Cho, Andy W. C. Pang, Mahendra Rao, Han Cao, Nicholas J. Schork, Jeanne F. Loring

**Affiliations:** 1 Scripps Translational Science Institute and The Scripps Research Institute, Department of Molecular and Experimental Medicine. La Jolla, California 92037. USA; 2 The Scripps Research Institute, Department of Chemical Physiology, Center for Regenerative Medicine, La Jolla, California 92037 USA; 3 BioNano Genomics, San Diego, California 92121. USA; 4 The National Institutes of Health, Bethesda, Maryland 20892. USA

## Abstract

There is concern that the stresses of inducing pluripotency may lead to deleterious DNA mutations in induced pluripotent stem cell (iPSC) lines, which would compromise their use for cell therapies. Here we report comparative genomic analysis of nine isogenic iPSC lines generated using three reprogramming methods: integrating retroviral vectors, non-integrating Sendai virus and synthetic mRNAs. We used whole-genome sequencing and *de novo* genome mapping to identify single-nucleotide variants, insertions and deletions, and structural variants. Our results show a moderate number of variants in the iPSCs that were not evident in the parental fibroblasts, which may result from reprogramming. There were only small differences in the total numbers and types of variants among different reprogramming methods. Most importantly, a thorough genomic analysis showed that the variants were generally benign. We conclude that the process of reprogramming is unlikely to introduce variants that would make the cells inappropriate for therapy.

## Abstract (web summary)

It is feared that reprogramming may introduce DNA mutations. Here Bhutani *et al*. take three different reprogramming methods and using comparative whole genome analyses do identify nucleotide variations that are different in reprogrammed cells from the original fibroblasts, but none convey oncogenic potential.

## Graphical abstract

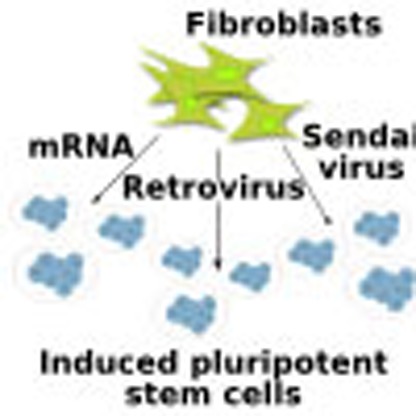

Cell replacement therapies using cells derived from human pluripotent stem cells (embryonic stem cells (hESCs) and induced pluripotent stem cells (iPSCs)) have been approved for clinical trials for macular degeneration, spinal cord injury and type-1 diabetes. The limited data available to date indicate that there are no adverse events in patients receiving these cells^1^. However, there continues to be discussion about the theoretical chance that these transplanted cells may develop into tumours or cause other pathologies. The discussion has come to focus on iPSCs, largely due to concerns that the massive epigenetic remodelling that occurs during reprogramming might cause genomic mutations that could make the cells tumorigenic. These concerns have led multiple groups to study the genomic integrity of iPSCs using methods that include single-nucleotide polymorphism (SNP) genotyping^1^,^2^, CGH^3^, karyotyping^4^ and exome sequencing^5^ (reviewed in refs 6, 7). In each case, the focus has been exclusively on a single type of genomic alteration, rather than considering the combined effects of single-nucleotide variants (SNVs), structural variants (SVs) and copy-number variations. Further, detailed comparative genomic analyses of iPSC lines that have been generated via distinct reprogramming methodologies have yet to be reported.

In this study, we assessed genome-wide mutation rates from replicate isogenic cell lines generated by three distinct methods. We used integrating viral (retrovirus), non-integrating viral (Sendai virus) and non-integrating non-viral (messenger RNA (mRNA)) reprogramming strategies to introduce exogenous expression of *POU5F1*, *SOX2*, *KLF4* and *MYC* in separate fractions of a single fibroblast population (Fig. 1a). Three clonal lines were established from iPSCs generated by each method and determined to be pluripotent by standard measures. To detect SNVs, SVs and copy-number variations within each line and the parental fibroblast population, we generated whole-genome sequencing data for each iPSC line and the parental fibroblasts at an average read depth of 39-fold, with 93.7% of the autosomal genome covered by at least 10 reads. In addition, to assess chromosomal rearrangements and large SVs with high resolution, we performed whole-genome mapping using the recently developed Irys Technology (BioNano Genomics, San Diego, CA). We detected subtle differences in the numbers of variants depending on the method, but rarely found mutations in genes that have any known association with increased cancer risk. We conclude that mutations that have been reported in iPSC cultures are unlikely to be caused by their reprogramming, but instead are probably due to the well-known selective pressures that occur when hPSCs are expanded in culture.

## Results

### Identification of single-nucleotide variants

**Figure 1:**
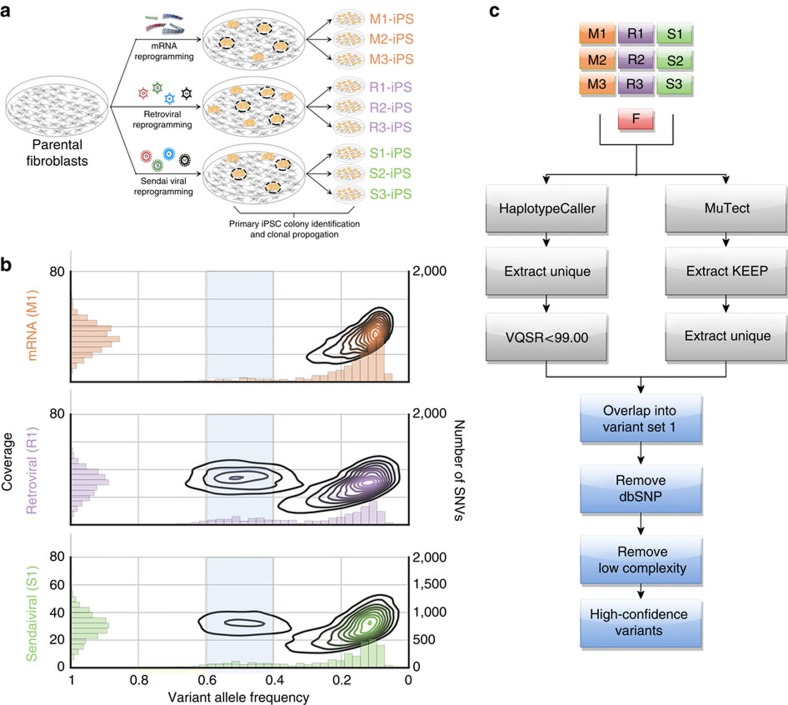
Experimental and computational design for identifying variants caused by reprogramming. (**a**) Diagram describing the derivation of three biological replicates of each three reprogramming methods: retrovirus, Sendai virus and non-integrating mRNA. (**b**) Kernel density estimation for VAF and coverage for a constituent sample from each reprogramming method: M1 (mRNA), R1 (retrovirus) and S1 (Sendai virus). For R1 and S1, there are denser clusters near 40 × coverage and 40–60% VAF than the M1 sample, which indicates they had a higher mutational load during initial doublings. However, it should be noted that all these samples also contained several subclonal variants that are not considered in further analyses. The histograms are intended to aid the readers in interpreting the results of the kernel density estimations. (**c**) Flow diagram detailing the filtering strategy employed to arrive at high-confidence set of SNVs unique to each reprogrammed cell line using MuTect and HaplotypeCaller.

To characterize the mutational burden in iPSCs, we identified SNVs that were unique to each iPSC cell line by integrating results from HaplotypeCaller^8^ and MuTect^9^, as described in the Methods section. For variant calling with HaplotypeCaller, we treated all 10 samples (the parent fibroblast population and three biological replicates for each reprogramming method) as part of a single population using the multisample option. This pipeline was tuned for the identification of recurrent variations in population studies, and therefore enabled us to have higher specificity in classifying reprogramming-induced mutations by accounting for mosaicism in the parental fibroblast population. To gain a more sensitive assessment of the SNV landscape across the iPSC samples, we also called variants using MuTect, wherein each iPSC line was compared with the parental fibroblast population in an analogous manner to which tumour samples are compared with normal tissue in oncogenomic studies (Fig. 1c; Supplementary Fig. 1). Taken together, the results from these two distinct variant calling pipelines gave us higher confidence in our ability to identify true variants through the moderation of type I and type II errors, respectively.

The identified set of putative unique variants was split into three groups according to our confidence in the variant calls: Variant Set 1 was called unique by both MuTect and HaplotypeCaller; Variant Set 2 had coverage between 20–60 × , and allele frequency distribution between 0.4 and 0.6 but was called only by MuTect, and Variant Set 3 comprised those with allele frequencies between 0.2–0.4 and 30–50 × coverage.

Variant validation by quantitative PCR from each of these three groups indicated that the mutations from Variant Set 1 had the highest likelihood of being true somatic variants (Supplementary Data 1; Methods). Therefore, subsequent analyses were restricted to these variants, which likely occurred during the initial doublings of the founder populations of the iPSCs compared with variants that are in lower allelic fractions that could arise as a result of proliferation of the cells. Our filtering strategy focuses on these high-confidence variants and also removes variants that are found in low-complexity regions and dbSNP (Fig. 1c; Supplementary Data 2).

### Identification of insertions and deletions

Due to the specificity of multisample HaplotypeCaller in identifying high-confidence SNVs, HaplotypeCaller was also used for identifying insertions and deletions (indels).

### Identification of structural variations

Structural variations (SVs), 10 kbp to 1 Mbp in size, are common in the human genome, but challenging to assess by sequencing alone. Genome mapping in nanochannel arrays provides a single-molecule platform complementary to DNA sequencing for accurate genome assembly and SV analysis (Irys System, BioNano Genomics)^10^,^11^,^12^,^13^. Unamplified genomic DNA, fluorescently labelled at a seven-base sequence motif and by the intercalating dye YOYO-1, was linearized by electrophoresis into 50-nm channels. An array of approximately 12,000 nanochannels was imaged for each sample, and the process was repeated multiple times, producing data at a throughput of about 1.5 Gbp h^−1^. Only molecules 150 kbp and longer were used to create a *de novo* assembly of the complete genome. We analysed the fibroblast control and one iPSC line chosen at random from each of the three reprogramming methods.

We collected 160 Gbp (∼50 × ) of high-molecular-weight DNA for each sample, ∼500,000 single-molecule maps with the minimal length of 150 kbp. Data from each sample were assembled using an in-house developed *de novo* assembler based on Overlap-Layout-Consensus paradigm^14^,^15^,^16^. The N50 length is the length for which the collection of genome maps of that length or greater cover >50% of the total genome length. The genome maps in this study had a *N50* of >0.9 Mbp, overlapping close to 90% of the Genome Reference Consortium Human genome (GRch37; Methods; Supplementary Fig. 2; Supplementary Table 1).

### Assessing pathogenicity of unique variants

**Figure 2:**
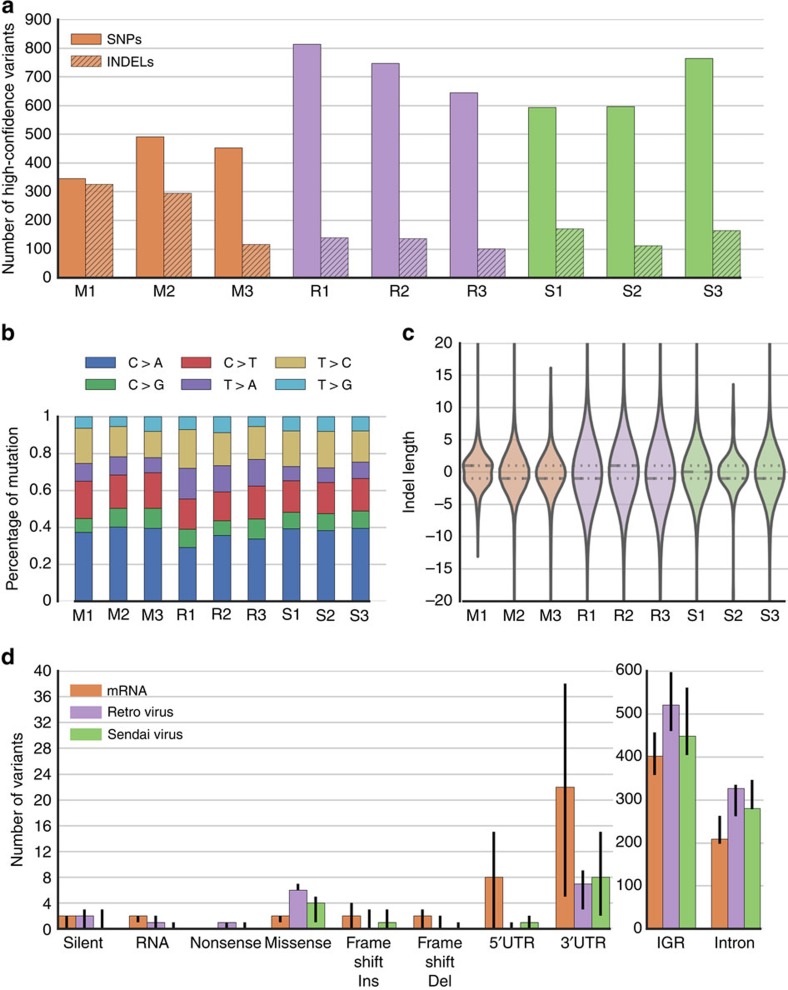
Characterization of variants caused by reprogramming method. (**a**) Overall counts for the number of high-confidence SNVs and indels per sample. (**b**) The relative percentage of mutational subtypes for the SNVs in each sample. (**c**) A violin plot and box plot for the indel size distributions in the sample, a positive length indicates an insertion, whereas a negative one is a deletion. (**d**) Variant classifications based on their relative locations in the genome. The error bars indicate the low, median and high replicate for each reprogramming method. Introns and IGR variants are plotted on a different scale.

To assess the functional consequences of the variants, the sets of high-confidence unique SNVs and indels were annotated using the SGAdviser^17^ and Oncotator^18^ program suites. They were further characterized based on overlaps with ENCODE annotated genomic regions. As expected, most mutations fell within intergenic or intronic regions, with the rate of coding mutations in the range of 2–10 mutations per cell line (Fig. 2d). The potential for variants to be oncogenic was assessed by measuring their overlap with cancer genes, transcription factor-binding sites (TFBS), MutSig genes and Familial Syndrome Cancer Genes (Methods; Supplementary Data 3). We then compared the frequency of the annotated somatic mutations across the three reprogramming methods using an exhaustive permutation testing procedure and one versus all contrasts in analysis of variance (ANOVA). All of the iPSC lines had hundreds of variants compared with the parental fibroblasts, with the mRNA-derived lines having fewer high-confidence mutations on average than the other methods (Fig. 2a; Tables 1 and 2; Supplementary Table 2). We did not find evidence that any one of the methods was more likely than the others to cause oncogenic or deleterious mutations, but we identified trends based on one versus all ANOVA contrasts that linked reprogramming strategies to certain mutations. The cell lines reprogrammed using mRNA had several variants that overlapped binding sites for a transcription factor (EZH2; enhancer of zeste 2 polycomb repressive complex 2 subunit), although it is unknown if these would disrupt transcription factor binding. The retroviral-induced cell lines harboured more potentially damaging mutations than the other methods (Table 3). In addition, the three retrovirally reprogrammed cell lines contained integrated vectors in 7, 8 and 12 sites in the genome, and while the majority of integration sites were intergenic, some integrations mapped to coding regions (Supplementary Fig. 3). In Sendai virus reprogrammed cell lines, there was a trend towards fewer coding mutations. However, we want to emphasize that based on the variance and means of the aggregated statistics, this study was not sufficiently powered to assess the differences among the different reprogramming methods for some variant classifications (Table 1).

We also characterized the context of the mutations, as well as the overall transition/transversion rates for the different samples (Fig. 2b). Mutational context analysis revealed no realizable difference among the different reprogramming methods and identified no links to known cancer-related mutational signatures^19^. Finally, we looked at the Combined Annotation-Dependent Depletion (CADD)^20^ score distribution of the variants, and although we saw statistically significant differences among the three methods, the scores for all methods fell mostly within the non-deleterious range of less than 15 CADD Score (Supplementary Fig. 4).

**Figure 3:**
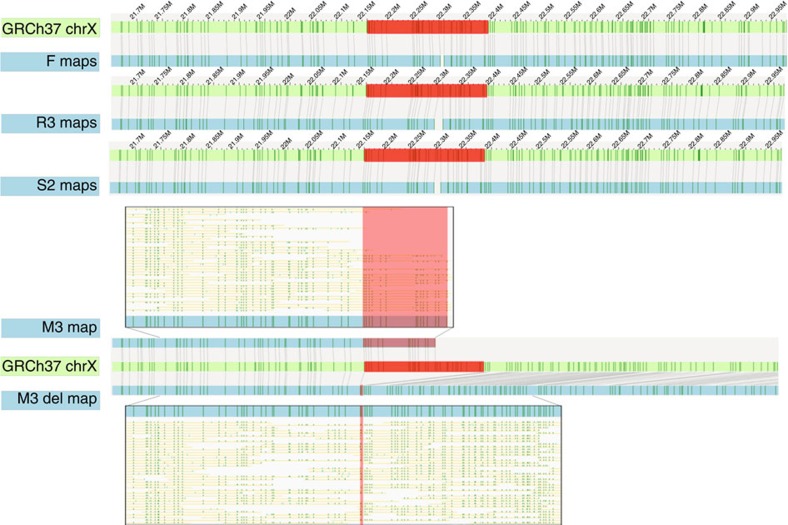
A 228.8-kb deletion at Xp22.11 in sample M3 detected by BioNano genome mapping. Each assembly is compared with the GRCh37 reference genome. Black vertical marks show the position of the fluorescently labelled seven-base motif. For the M3 sample, observed individual DNA molecules and their labels are represented, showing the support for two haplotypes, one with the deletion at Xp22.11.

Using the variant calling algorithms for the data from *de novo* whole-genome mapping of four of the samples, we called 259 insertions and deletions in the parental fibroblast sample, 239 in the retroviral sample, 248 in the Sendai sample and 268 in the mRNA sample. The size of the variants ranged from 2.8 kbp to 4.9 Mbp. Using a 50% reciprocal size overlap cutoff, we identified variants that were shared among the samples. After manual inspection to eliminate false positives and false negatives, we found no cell line-specific variants in the retroviral and Sendai samples, but one deletion in the mRNA-reprogrammed sample. The deletion in this line was a heterozygous 228.8-kbp deletion at Xp22.11, which removed one copy of the *PHEX* gene (phosphate-regulating neutral endopeptidase) and one copy of Mir_548. This deletion is illustrated in Fig. 3, which shows the assembly of part of the X chromosome for each cell line, as well as the single-molecule data supporting two haplotypes for this region in the mRNA sample. We subsequently looked for this deletion in the sequencing data and identified it in the same sample; it was not present in any of the other samples. This suggests that this iPSC line was derived from a rare fibroblast containing the deletion or that the deletion was acquired very early in the reprogramming process.

## Discussion

Our assessment of the mutation profiles associated with three widely used reprogramming methods for generating iPSCs indicates that all of the reprogramming approaches add to the mutational load of cells, but there were subtle differences among the methods. Although we found that the non-integrating mRNA reprogramming technique resulted in fewer total mutations than either retrovirus or Sendai virus-based reprogramming methods, mRNA-iPSCs had a greater number of mutations in binding sites for a transcription factor (EZH2). In addition, the only large structural variation (a 228.8-kbp deletion) we detected was in an mRNA-reprogrammed iPSC line. In contrast, the Sendai virus samples had fewer coding mutations than the other methods.

Our results using retroviral vectors showed that this method caused a similar number of mutations as the other methods, but was slightly more likely to introduce mutations that are classified as deleterious. But the main concern with retroviral vectors is the fact that they insert into the genome. Genomic insertions have a low but finite chance of disruption of active genes or regulatory regions, and can cause cancers if they activate endogenous oncogenes^21^. Retroviral vectors were used in the first methods developed for reprogramming^22^ but were reported to be capable of reactivation, which resulted in tumours in mice^23^. Use of retroviral vectors for reprogramming has become less popular as delivery methods for transiently expressing the reprogramming factors without being inserted, such as the Sendai viral vectors and mRNA used here, have become more efficient.

Given the potential practical significance of our findings to clinical applications for stem cells, it is important to appreciate some of the biological context surrounding our experiments. We focused on the high-confidence variants that differed from the parental fibroblast population. These variants likely arose during the initial doublings of the founder population of iPSCs, but we cannot rule out their origin in a minority population in the heterogeneous parental fibroblasts that was undetectable by sequencing. We note that there were sequencing reads that support several additional lower variant allele frequency mutations in the cell lines as well. It should be noted that the cells we analysed were cultured for a relatively short time, and that variants in an early-arising small subpopulation could become more dominant over time if they give the cells a selective advantage in culture^6^.

To move forward towards applying stem cell-based therapies for human disease, it is important to focus our efforts on improving the likelihood that there will be no adverse effects of these therapies. Our study was more extensive than previous analyses, but larger studies of this depth are still needed. While results of our study do not rule out the possibility that reprogramming cells could introduce oncogenic mutations that compromise the safety of iPSCs, they should alleviate some of the concern about how likely it is that reprogramming itself would cause dangerous genomic changes that could lead to harm to transplant recipients. It is important to note that genomic aberrations, some of which could be oncogenic, are known to occur during the considerable expansion of cells that is required for clinical applications. While development of new methods for reprogramming will make the process simpler and less expensive, it is critical at this stage that we concentrate on monitoring the appearance and potential consequences of mutations that arise during cell division and differentiation in culture and are selected for by the culture conditions.

## Methods

### Overview of experimental approach

The design of our study was to evaluate mutation profiles associated with the three different reprogramming strategies (see below for details on each method) we considered, as well as the characterization of different forms of variation and the analysis of the variation within and across the different reprogramming strategies.

### Retroviral reprogramming

PLAT-A-packaging cells (Cell Biolabs, Inc.) were plated onto six-well plates coated with poly-D-lysine at a density of 1.5 × 10^6^ cells per well without antibiotics and incubated overnight. Cells were transfected with 4 μg of Moloney murine leukaemia-based retroviral vectors (pMXs) containing the human complementary DNA of *POU5F1*, *SOX2*, *KLF4* or *MYC* (Addgene catalog number 17217, 17218, 17219, and 17220, respectively) by Lipofectamine 2000 (Life Technologies, Carlsbad, CA) according to the manufacturer's instructions. Viral supernatants were collected at 48 and 72 h post transfection, filtered through a 0.45-μm pore-size filter. 200,000 Human dermal fibroblasts (Science cell Catalog number 2300) were seeded onto each well of a six-well plate overnight prior transfection. Equal volumes of fresh 48 and 72 h viral supernatants containing each of four retroviruses supplemented with 6 μg ml^−1^ of Polybrene (Sigma) were added onto the cells on day 1 and day 2, respectively. On day 5, the transduced cells were split onto mouse embryonic fibroblasts (MEFs) at a density of 10^4^ cells per well of a six-well plate in hESC medium supplemented with 0.5 mM valproic acid (VPA; Stemgent). Cells were fed every other day with VPA-supplemented hES medium for 14 days before VPA was withdrawn. Individual iPSC colonies were manually picked and clonally expanded 3 weeks post transduction and transferred onto MEF plates.

### Sendai virus reprogramming

Human dermal fibroblasts (Science cell Catalog number 2300) were reprogramed according to the manufacturer's instructions (CytoTune-iPS 2.0 Sendai Reprogramming Kit, Life technology catalog number A1378001). Cells were transduced with Sendi viruses containing the Yamanaka factors, and individual iPSC colonies were identified by morphology. The colonies were manually picked, expanded for 3 weeks post transduction and transferred onto a feeder layer of irradiated MEFs.

### mRNA reprogramming

Human dermal fibroblasts (Science cell Catalog number 2300) were reprogramed according to the manufacturer's instructions (The Stemgent mRNA Reprogramming Kit catalog number 00–0071). Individual iPSC colonies were manually picked and clonally expanded three weeks post transfection and transferred onto MEF feeder layers.

### Cell culture

Plat-A-packaging cells (Cell Biolabs, Inc.) were maintained according to the manufacturer's instructions. Human dermal fibroblasts (Science cell Catalog number 2300) were cultured in DMEM, 2 mM GlutaMax, 10% fetal bovine serum and 0.1 mM nonessential amino acids (Life Technologies). iPSCs were generated and maintained in standard hESC medium containing DMEM/F12 supplemented with 20% Knockout Serum Replacement (Life Technologies), 2 mM GlutaMAX, 0.1 mM nonessential amino acids, 0.1 mM 2-mercaptoethanol and 12 ng ml^−1^ of Human Recombinant Fibroblast Growth Factor-basic (bFGF, Stemgent). HDFiPS cells were cultured on MEF feeder layers in hESC medium and mechanically passaged once a week. The hESC medium was changed daily. All cultures were tested and were negative for mycoplasma.

### DNA extraction and sequencing

The Qiagen DNeasy Blood and Tissue Kit (catalog number 69504) was used to prepare genomic DNA from ∼2 million cells of each cell line, as recommended by the manufacturer. Template DNA fragments (3 μg) were hybridized to the surface of flow cells HiSeq Paired-End cluster Kits (v2.5 or v3) and amplified to form clusters using the Illumina cBot. Paired-end libraries were sequenced for 2 × 101 cycles of incorporation and imaging using TruSeq SBS kits. Sequencing was performed at Illumina, Inc. (San Diego).

### Realignment of illumina reads and recalibration

Reads were extracted from Illumina Casava aligned BAM files using the HTSLib by first shuffling the reads and then extracting interleaved reads; see http://www.broadinstitute.org/gatk/guide/article?id=2908. These reads were then processed through the GATK Best Practices workflow for Variant Calling v.2.6, which included first aligning the reads using BWA 0.7 with the ‘mem' option, marking duplicates, pursuing local realignment, considering base quality recalibration and finally using the ‘reduce reads' options. The BAM files generated from this process were used with the different variant calling methods.

### SNVs and indel variant calling

HaplotypeCaller, as bundled with GATK v2.7, was used to call all 10 samples together. The variant calls were recalibrated using files in the GATK bundle that included data from HapMap, Omni, dbSNP, Mills Indels and 1,000 Genomes Indels databases. Variants falling in tranche levels 0–90 and 90–99.00 were used for downstream analysis. Unique variants were identified by looking at positions where only one sample had a non-reference allele.

It should be noted that the HaplotypeCaller multisample calling pipeline described sacrifices sensitivity for specificity, as it is meant for population scale studies. The pipeline may favour the identification of variants common across the samples rather than variants or mutations unique to each. To gain a more sensitive assessment of the somatic SNVs landscape across the iPSC samples, we also ran MuTect by treating the parent fibroblast population as the normal and each iPSC cell line as a different derived cell (in an analogous manner to which tumours are treated with respect to germline samples in oncogenomics studies). MuTect was run with default settings and the calls were filtered using the judgment ‘KEEP' option. Unique variants from the analysis were determined by intersecting the calls based on chromosomal coordinates and the variant calls.

### SNVs and indel validation and filtering

To validate the identified variants, we split them into three groups of confidence: high-confidence variants had coverage between 40–60 × with the variant allele frequency in the range of 0.4–0.6; low-confidence variants were classified as having coverage 20–40 × with variant allele frequency between 0.4–0.6; and subclonal variants were those with variant allele frequencies between 0.2–0.4 and 40–60 × . We validated these results using quantitative PCR. The ABL files associated with the variants were read in using the ‘abifpy' package (https://github.com/bow/abifpy ), and looking at the calls made by the SOLID software. For any variants that appeared heterozygous, the relative amount of noise in the 10 upstream and downstream bases was evaluated based on the assumption that they should be homozygous. Any amplitude values that were two s.d.'s higher than the mean background noise were called as variant calls. Most of the calls made in this manner were validated through manual inspection (Supplementary Table1).

### SNVs and indel variant annotation

The variants were run through the SGAdviser (http://genomics.scripps.edu/ADVISER) pipeline for annotation. A SNV was considered damaging if it had a harmful designation by Condel, Polyphen or SIFT. To assess the likely oncogenic potential of any variant, we assessed overlaps with the MKCC Cancer Genes, Atlas Oncology and Sanger Cancer Genes. Variants that overlapped TFBSs were assessed as being damaging by looking at changes calculated by the MOODS algorithm^24^. High-confidence TFBS altering mutations were those that changed the binding affinity by more than 7. We also ran the variants through the Onconator annotation web service (http://www.broadinstitute.org/oncotator/) and found overlaps with MutSig genes and Familial Syndrome Cancer Genes. Finally, the variants were fed through CADD annotation service through their online web service.

### ENCODE TFBSs annotation

Genomic coordinates (hg19) for transcription factor-bound regions of DNA, as curated by ENCODE (wgEncodeRegTfbsClusteredV3.interval ), were downloaded from the UCSC genome browser Main Page (via Galaxy) in *.bed format. In addition, the genomic coordinates (hg19) for all iPSC variants (SNVs/indels) were concatenated into a single bed file and intersected with the transcription factor genomic intervals (bedtools) to determine whether specific transcription factor-binding profiles were disproportionally enriched with/depleted of mutations in iPSCs generated via one reprogramming method in comparison with the others.

### Identification of integration sites

Integration sites were identified by looking at the paired-end reads in which one-end mapped to the reference genome hg19 and one-end maps to the viral sequences. The one-end mapped reads were extracted from the BAM files using the command samtools view^25^, and then aligned to the viral genomes using BWA v. 0.7 aligner^26^. The ‘readnames' identifiers were used to find the pair in the hg19 aligned reads. These were further filtered down to require at least five reads matching on either end of the integration site and were annotated by intersecting the ‘knownGenes' track on UCSC.

### Permutation testing

We leveraged permutation testing in an ANOVA setting to find the probability of observing the differences in mutation and variant rates across the three reprogramming methods. We exhaustively relabelled all the reprogramming types, recomputing the ANOVA statistic each time we relabelled the cell lines, thus determining exactly how likely it would be to observe our mutation profile differences across the reprogramming cell types given the three replicates for each of the three reprogramming methods (a total of 280 permutations). We tested each genomic feature separately using this permutation strategy. To calculate the relative rates of different types of mutations (for example, coding, non-coding and so on), we divided by the total number of mutations.

### ANOVA contrasts

We also tested whether one method was different from the other two methods using contrasts. Three different tests were employed for mRNA versus all, retroviral versus all and Sendai virus versus all using the contrasts (−2, 1, 1), (1, −2, 1) and (1, 1, −2), respectively. We employed an ANOVA fit to estimate the impact of the reprogramming strategy on the aggregated counts of the different variant classifications. We employed this strategy to look at the difference in the nominal counts for the different variant classifications, but we also subdivided by the type of variants (SNV, insertion or deletion), as well as the relative rates of each variant type. We further filtered reported results to classifications that had at least 10 variants.

### Comparison of CADD score distributions

To compare the distribution of CADD Scores across the different samples, we employed a Kruskal–Wallis test. First, we looked at the differences between the replicates of the same reprogramming method to ensure that the variance within a group was not high. Next, we looked at pair-wise comparisons between the different reprogramming methods by pooling all the variants for the replicates into one distribution for the reprogramming method. This analysis revealed that synthetic mRNA had a distribution skewed towards lower scores compared with the retroviral vectors and non-integrating Sendai virus. There was no realizable difference between the retroviral vectors and non-integrating Sendai virus. However, further inspection of the distributions revealed that most of the variants still fell in the non-damaging designation of CADD scores, indicating that the results were not biologically significant.

### BioNano high-molecule-weight DNA extraction

HDF51iPS11, HDF51iPS509, HDF51iPS1003 and HDF51 cell lines—termed R3, S3, M3 and F cell lines, respectively—were constructed on-site at BioNano Genomics, where they were washed with 1 × PBS, placed in resuspension buffer and embedded into agarose gel plugs (BioRad, Hercules, CA). Embedded cells were incubated with lysis buffer (BioNano Genomics) and proteinase K for four hours at 50 °C. Agarose was solubilized with GELase (Epicentre, Madison, WI) and extracted DNA was drop dialyzed for four hours. DNA concentrations were measured using the Quant-iT dsDNA Assay Kit (Life Technologies).

### Bionano DNA labelling

DNA was labelled following the IrysPrep Reagent Kit protocol (BioNano Genomics). Briefly, 900 ng of DNA was digested with 10 U of Nt.BspQI nicking endonuclease (New England BioLabs, Ipswich, MA) for 2 h at 37 °C. Nick digested DNA was then incubated for 1 h at 72 °C with fluorescently labelled dUTP and Taq Polymerase (New England BioLabs). Taq ligase (New England BioLabs) was used in the presence of dNTPs for ligation of nicks. DNA was counterstained with YOYO-1 (Life Technologies).

### BioNano data collection

Labelled and counterstained DNA samples were loaded into IrysChips (BioNano Genomics) and run on the Irys (BioNano Genomics) imaging instrument. Data were collected for each sample until ≥50-fold coverage of long molecules (>150 kbp) was achieved. The IrysView (BioNano Genomics) software package was used to detect individual linearized DNA molecules using the YOYO-1 counterstain and to determine the localization of labelled nick sites along each DNA molecule. Sets of single-molecule maps, equivalent to ∼50 × haploid coverage, for each sample were then used to build a full-genome assembly.

### BioNano *de novo* assembly

*De novo* assembly of single molecules is accomplished using BioNano's custom assembler software program based on an Overlap-Layout-Consensus paradigm^13^,^14^,^15^,^16^. First, we started with pair-wise comparison of all molecules longer than 150 kbp and ≥5 labels to find all overlaps with a *P* value<5 × 10^−10^, and then we could construct a draft consensus genome map based on these overlaps. The draft map could be further refined by mapping single molecules to it and recalculating the label positions. Next, the maps were extended by aligning overhanging molecules to the maps and calculating a consensus in the extended regions. Finally, the genome maps were compared and merged where patterns match with a *P* value<10^−15^. The process of extension and merge was repeated five times before a final refinement was applied to ‘finish' all genome maps. The result of this assembly is a genome map set entirely independent of any known reference or external data (Fig. 3). Statistics about N50 and percentage coverage of GRCh37 are described in Supplementary Table 1.

### BioNano structural variation calls

Structural variation was detected by examining the alignment profiles between the *de novo* assembled genome maps against the GRCh37 human reference assembly. Significant discrepancies in (a) the distance or (b) the number of unaligned labels between adjacent aligned labels would indicate the presence of insertion and deletion events. We used two algorithms to call SV, and they differ in the way discrepant regions in the alignment (termed outliers) were handled. In the first algorithm, the reference and maps were split at outliers, and split maps were iteratively realigned. The alignments of the newly split maps would then pinpoint the locations of the insertion and deletion variants. We used an alignment *P* value of 10^−12^ and an outlier cutoff of 10^−4^ to call variants in all four cell line samples. In the second algorithm, the reference and genome maps were not split; instead, the global alignment profiles were kept with insertions and deletion events being intra-alignment gaps. For all four cell line samples, the alignment *P* value was 10^−12^ and the outlier cutoff was 10^−4^. Alignment *P* values are calculated using the algorithm described in Anantharaman *et al*.^14^.

### BioNano identification of cell line-specific calls

A series of steps were used to identify cell line-specific variants. First, we conservatively selected only insertions and deletions detected by both calling algorithms. Using a 50% reciprocal size overlap cutoff, we cross-compared variants detected among all cell lines to identify those that were putatively cell line specific. Finally, we manually curated the candidate variants to ensure that (a) there were molecules supporting the variant allele in the cell line of interest and (b) there was no molecule supporting the variant allele in all other cell line samples.

## Additional information

**Accession codes:** The whole-genome sequencing data have been deposited in the NCBI database under accession codes: BioProject ID: PRJNA304745; SRA database: SRP067962. The BioNano Genomics mapping data are available at http://bnxinstall.com/publicdatasets/ipsc_Bhutani_et_al_2015.zip

**How to cite this article:** Bhutani, K. *et al*. Whole-genome mutational burden analysis of three pluripotency induction methods. *Nat. Commun.* 7:10536 doi: 10.1038/ncomms10536 (2016).

## Supplementary Material

Supplementary Information Supplementary Figures 1-4 and Supplementary Tables 1-2

Supplementary Data 1 The noise and normal peaks for qPCR amplified positions.

Supplementary Data 2 List of all high confidence variants.

Supplementary Data 3 Aggregated statistics for assessing the potential pathogenicity of high confidence variants.

## Figures and Tables

**Table 1:** *P* values from the permutation-based ANOVA test for variant-type differences across the three reprogramming methods.

	SNV raw counts	SNV relative rates	Insertions raw counts	Deletions raw counts	Indel raw counts	Combined	Samples for 80% power
CADD Phred>15	0.12	0.36	ND	ND	ND	0.12	4
Coding	0.03	0.18	0.34	0.23	0.24	0.16	4
Damaging	0.09	0.30	ND	ND	ND	0.09	4
Near cancer gene	0.10	0.94	0.11	0.20	0.14	0.39	10
Total	0.01	ND	0.18	0.16	0.16	0.20	6

ANOVA, analysis of variance; CADD, Combined Annotation-Dependent Depletion; ND, not determined; indels, insertions and deletions; SNV, single-nucleotide variant.

Rates were determined by dividing the number of variants by the total number of variants. ND either because it is not consistent with the calculations or there were too few variants to analyse. Combined is the sum of SNVs and indels. The last column lists the sample size estimates necessary based on 80% power for an ANOVA statistic given the current mean and variance for the grouping by reprogramming methods. This is based on the combined counts.

**Table 2:** High-confidence variants in coding regions.

	Number of high-confidence variants		
Sample	Synonymous	Nonsense and nonsynonymous	Coding regions affected by high-confidence nonsense and nonsynonymous variants
M1	0	2	Nonsynonymous: *BC068088* and *C14orf159*
M2	2	2	Nonsynonymous: *BPIFB1* and *MACROD1*
M3	2	1	Nonsynonymous: *RPAP2*
R1	2	7	Nonsense: *SALL1*; Nonsynonymous: *C2orf91*, *CCDC150*, *SSC5D*, *SYT4*, *UTRN* and *WDR72*
R2	0	7	Nonsense: *PRR12*; Nonsynonymous: *ADAM18*, CATSPERG*, IKBIP, *NCR3LG1*, *PRR12* and *SPTA1*
R3	3	7	Nonsynonymous: *ALPI*, *HIST1H2BD*, *ITGB8*, *OR5AP2*, *PKP2*, *RNF10* and *TMPRSS5*
S1	0	5	Nonsynonymous: *KCNC3*, *OVOS2*, *SDR16C5*, *XPR1* and *ZNF660*
S2	3	4	Nonsynonymous: *CLEC5A*, *FAM208B*, *MMEL1* and *PCDHB16*
S3	0	2	Nonsense: *FSCN3;* Nonsynonymous: *FSCN3* and *PRDM4*

The number of high-confidence synonymous and nonsynonymous coding mutations identified with high-confidence SNVs in each sample. Nonsynonymous variants in protein-coding regions are listed. M1-3: mRNA vector; R1-3: Retrovirus; S1-3: Sendai virus.

^*^The *CATSPERG* gene has two nonsynonymous high-confidence mutations in this sample.

**Table 3:** Functional impacts as calculated by ANOVA contrasts using a one versus all approach.

	M1	M2	M3	R1	R2	R3	S1	S2	S3	*P* value
Non-integrating mRNA versus all others—transcription factor EZH2-binding sites										
	13	11	6	1	7	2	3	0	3	0.008
										
Retrovirus versus all others—damaging mutations assessed by Condel, Polyphen or SIFT										
	5	3	1	5	9	13	5	3	2	0.014
										
Sendai virus versus all others—mutations in coding regions										
	27	16	9	16	20	24	12	9	5	0.044

ANOVA, analysis of variance; mRNA, messenger RNA

ANOVA contrasts were set-up to compare one reprogramming methods against the other two. For each reprogramming method, the most significant difference is presented. The EZH2-binding site overlaps were determined by ENCODE annotations (Methods).
