# Supplementary material for: Whole-genome mutational burden analysis of three pluripotency induction methods
Source: Nat Commun. 2016 Feb 19;7:10536. doi: 10.1038/ncomms10536 (PMC4762882; doi:10.1038/ncomms10536)
Supplement: Supplementary Information — Supplementary Figures 1-4 and Supplementary Tables 1-2 [file ncomms10536-s1.pdf]

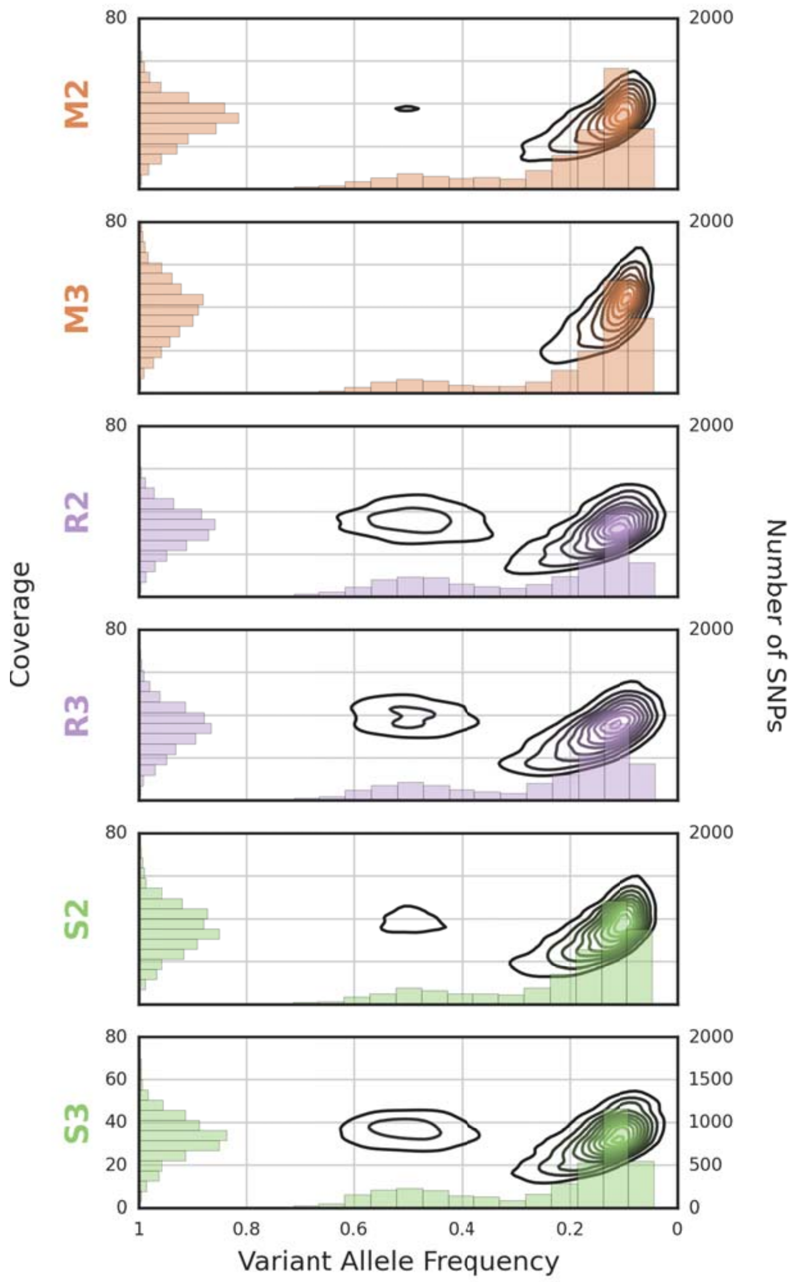

ss

Supplementary Figure 1: Kernel Density Estimation of Coverage and VAF of variants unique to cell lines. Kernel density estimation based on variant allele frequency (VAF) and coverage for samples not presented in main text Figure 1.

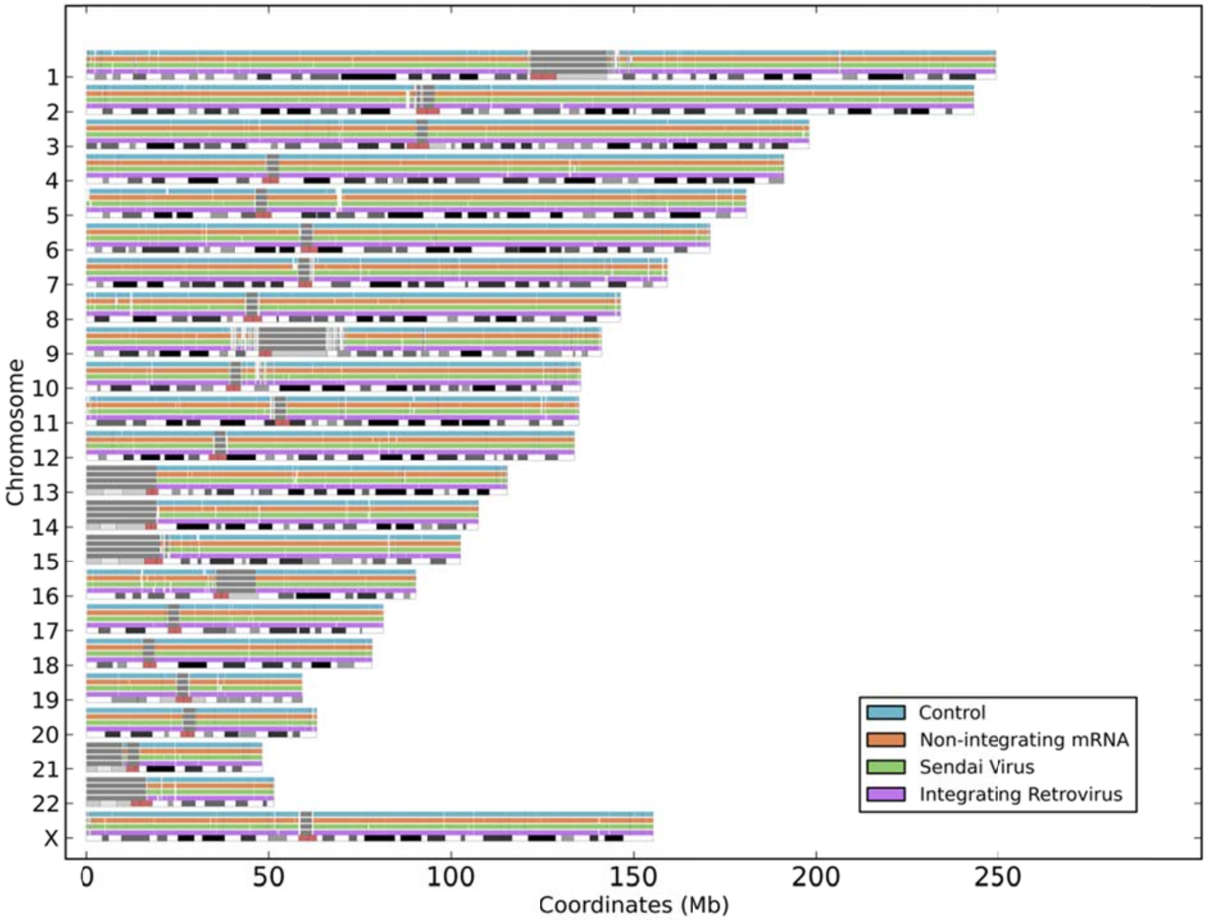

Supplementary Figure 2*: De novo* assemblies of the fibroblast control and one of each iPSC type from nanochannel mapping data. Genome maps of the four cell lines were aligned to the GRch37 reference map. Ideogram and Giemsa banding is plotted at the bottom of each chromosome in grey scale, with centromeres highlighted in light red. In each de novo assembly, white spaces separate contigs, and "N" base gaps in the reference are shaded with grey.

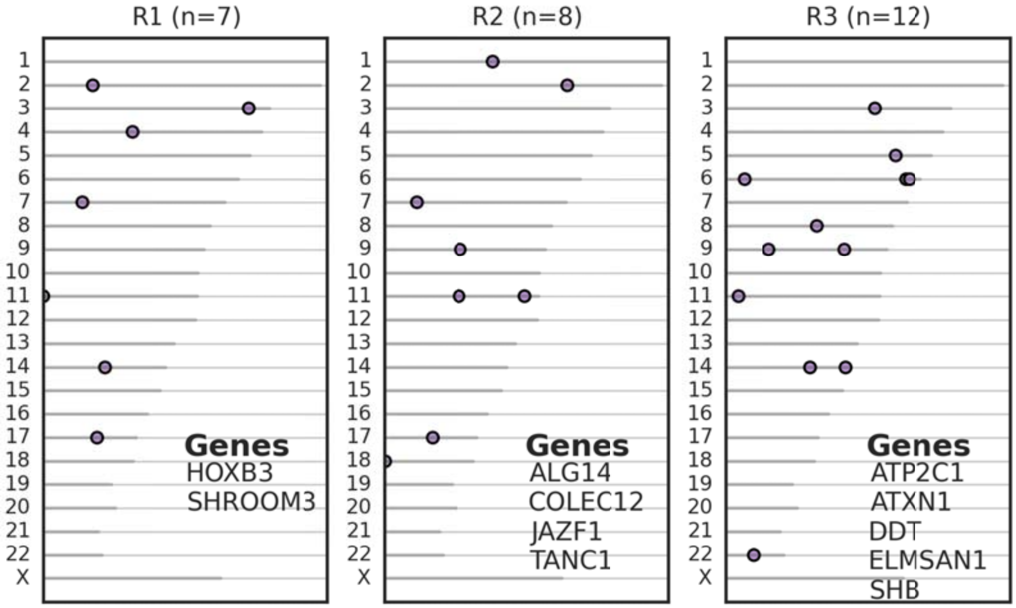

Supplementary Figure 3: Integration Sites of Retrovirus in the three replicates Integration sites in the three retroviral induced cell lines based on the methods outlined in the Methods. A list of genes is also provided for integration sites that were not intergenic.

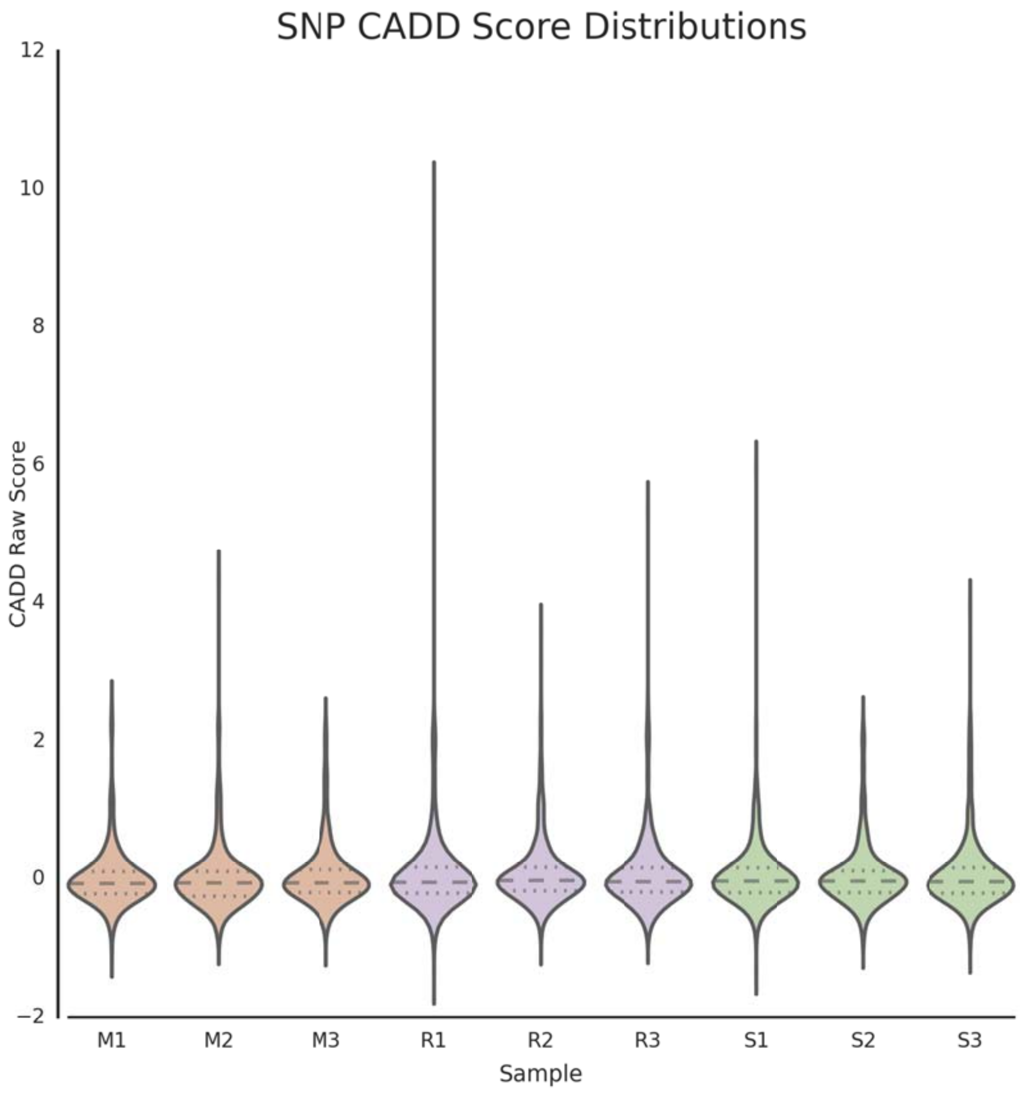

Supplementary Figure 4: The distribution of CADD scores for each sample. To investigate the potential pathogenicity of the variants, we compared the CADD scores of SNVs across the three different reprogramming methods. Although there is a statistical difference between the reprogramming methods (Kruskal-Wallis p-value 0.02) the results are biologically insignificant based on the criteria that most known damaging SNPs fall above a CADD Score of 15.

Supplementary Table 1: Statistics of molecules and de novo assembled genome maps.

	Control (F)	Integrating retrovirus (R3)	Non-integrating Sendai virus (S2)	Non-integrating mRNA (M3)
**Molecule N50 (kb)**	284.15	261.35	259.91	255.55
**Molecule Coverage (X)**	50.71	51.91	51.91	51.90
**Assembly Genome Maps (#)**	3,886	3,832	3,930	3,799
**Total Genome Map Len (Mb)**	2,855.30	2,820.79	2,829.19	2,847.77
**Genome Map N50 (Mb)**	0.93	0.95	0.90	0.97
**Total Genome Map Len / Ref Len**	0.92	0.91	0.91	0.92
**Total Unique Len / Ref Len**	0.89	0.89	0.88	0.89

Supplementary Table 2

The number of synonymous and non-synonymous coding mutations identified in each sample using MuTect.

Sample	Number of variants identified by MuTect	
	Synonymous	Nonsense and nonsynonymous
M1	92	32
M2	133	42
M3	24	20
R1	8	13
R2	25	20
R3	7	21
S1	44	17
S2	31	20
S3	7	9
